# Radiomics analysis for prediction of lymph node metastasis after neoadjuvant chemotherapy based on pretreatment MRI in patients with locally advanced cervical cancer

**DOI:** 10.3389/fonc.2024.1376640

**Published:** 2024-05-08

**Authors:** Jinjin Liu, Linxiao Dong, Xiaoxian Zhang, Qingxia Wu, Zihan Yang, Yuejie Zhang, Chunmiao Xu, Qingxia Wu, Meiyun Wang

**Affiliations:** ^1^ Department of Medical Imaging, People’s Hospital of Zhengzhou University (Henan Provincial People’s Hospital), Zhengzhou, Henan, China; ^2^ Department of Medical Imaging, People’s Hospital of Henan University (Henan Provincial People’s Hospital), Zhengzhou, Henan, China; ^3^ Department of Radiology, the Affiliated Cancer Hospital of Zhengzhou University, Henan Cancer Hospital, Zhengzhou, Henan, China; ^4^ Beijing United Imaging Research Institute of Intelligent Imaging, United Imaging Intelligence Co., Ltd., Beijing, China; ^5^ Laboratory of Brain Science and Brain-Like Intelligence Technology, Institute for Integrated Medical Science and Engineering, Henan Academy of Science, Zhengzhou, Henan, China

**Keywords:** magnetic resonance imaging, radiomics, cervical cancer, neoadjuvant chemotherapy, lymph node metastasis

## Abstract

**Background:**

This study aims to develop and validate a pretreatment MRI-based radiomics model to predict lymph node metastasis (LNM) following neoadjuvant chemotherapy (NACT) in patients with locally advanced cervical cancer (LACC).

**Methods:**

Patients with LACC who underwent NACT from two centers between 2013 and 2022 were enrolled retrospectively. Based on the lymph node (LN) status determined in the pathology reports after radical hysterectomy, patients were categorized as LN positive or negative. The patients from center 1 were assigned as the training set while those from center 2 formed the validation set. Radiomics features were extracted from pretreatment sagittal T2-weighted imaging (Sag-T2WI), axial diffusion-weighted imaging (Ax-DWI), and the delayed phase of dynamic contrast-enhanced sagittal T1-weighted imaging (Sag-T1C) for each patient. The K-best and least absolute shrinkage and selection operator (LASSO) methods were employed to reduce dimensionality, and the radiomics features strongly associated with LNM were selected and used to construct three single-sequence models. Furthermore, clinical variables were incorporated through multivariate regression analysis and fused with the selected radiomics features to construct the clinical-radiomics combined model. The diagnostic performance of the models was assessed using receiver operating characteristic (ROC) curve analysis. The clinical utility of the models was evaluated by the area under the ROC curve (AUC) and decision curve analysis (DCA).

**Results:**

A total of 282 patients were included, comprising 171 patients in the training set, and 111 patients in the validation set. Compared to the Sag-T2WI model (AUC, 95%CI, training set, 0.797, 0.722-0.782; validation set, 0.648, 0.521-0.776) and the Sag-T1C model (AUC, 95%CI, training set, 0.802, 0.723-0.882; validation set, 0.630, 0.505-0.756), the Ax-DWI model exhibited the highest diagnostic performance with AUCs of 0.855 (95%CI, 0.791-0.919) in training set, and 0.753 (95%CI, 0.638-0.867) in validation set, respectively. The combined model, integrating selected features from three sequences and FIGO stage, surpassed predictive ability compared to the single-sequence models, with AUC of 0.889 (95%CI, 0.833-0.945) and 0.859 (95%CI, 0.781-0.936) in the training and validation sets, respectively.

**Conclusions:**

The pretreatment MRI-based radiomics model, integrating radiomics features from three sequences and clinical variables, exhibited superior performance in predicting LNM following NACT in patients with LACC.

## 1 Introduction

Cervical cancer remains the fourth most common cancer and the fourth leading cause of cancer-related death among women worldwide (1–3). Locally advanced cervical cancer (LACC) refers to cervical cancer in stage IB3-IVA according to the 2018 International Federation of Gynecology and Obstetrics (FIGO) stage system (4). Lymph node metastasis (LNM) is an independent prognostic factor in LACC and affects the therapeutic decision (5). Both imaging and pathology findings can be utilized to assess pelvic and paraaortic lymph nodes (LN). Neoadjuvant chemotherapy (NACT), also referred to as early or prechemotherapy, serves the purpose of diminishing tumor volume, pelvic LNM rates, and parametrial infiltration rates before surgery or radiotherapy, thereby mitigating the surgical challenges (6, 7). NACT followed by radical hysterectomy has been proposed as an alternative approach of cisplatin-based chemotherapy and radiotherapy (CCRT) plus brachytherapy in patients with LACC (8, 9). It has been demonstrated that LNM post-surgery is also a significant factor for prognosis and treatment strategies in LACC patients who underwent NACT (10–12). Postoperative LACC patients with positive LN may necessitate adjuvant chemoradiotherapy (13). Therefore, accurately assessing LNM preoperatively for LACC patients is imperative for formulating effective treatment strategies, potentially preventing unnecessary surgical procedures and complications such as lymphatic exudation, lymphocele, lymphedema, neurovascular injury, wound infection et al (14, 15).

Magnetic resonance imaging(MRI) is the preferred method for pretreatment staging of cervical cancer (16). The diagnosis of LNM on MRI relies on morphological criteria such as size, shape, and texture. Nevertheless, the performance of direct MRI-based pelvic nodal assessment is relatively poor, with high specificity but low sensitivity (27%-45.7%) results in difficulty in detecting normal-sized, but positive LNs (17–19). Under this circumstance, radiomics, a form of image-based quantitative analysis, is becoming a current research orientation of imaging diagnosis, which can be used to quantify heterogeneity, predict outcome, and longitudinally monitor treatment responses (20). Recent evidence has shown that lesion-based radiomics features of preoperative MRI in LACC can improve the sensitivity of predicting LNM (21–24).

However, no studies have investigated the predictive value of pretreatment MRI features for LNM in LACC patients who undergo NACT. This study aims to create a model for predicting LNM after NACT based on pretreatment MRI.

## 2 Materials and methods

### 2.1 Patients

This retrospective study was approved by the institutional review board of center 1 (Henan Province People’s Hospital, HPPH) and center 2 (Henan Cancer Hospital, HCH), and the requirement for informed consent was waived.

We searched hospital information system with cervical cancer between January 2013 and February 2022, and found 380 consecutive cervical cancer patients who accepted a complete NACT regimen and radical hysterectomy with definite pathological results in two hospitals. According to postoperative pathological reports, patients were labeled as LN positive [LN (+)] and negative [LN (-)] groups. The inclusion criteria were as follows: (i) patients with FIGO stage IB3-IVA; (ii) patients who underwent pelvic MRI for pretreatment evaluation before NACT; (iii) Patients with a b-value of 800 s/mm^2^ on DWI; (iv) patients who received 1 to 3 cycles of NACT followed by hysterectomy and lymphadenectomy; (v) patients who had definite postoperative pathological results. The exclusion criteria were as follows: (i) patients with poor MRI quality; (ii)patients who were treated with NACT and brachytherapy or external beam radiotherapy simultaneously; (iii) patients who were judged to be inoperable after NACT and received CCRT; (iv) patients who underwent selective lymphadenectomy without hysterectomy after NACT.

**Figure 1:**
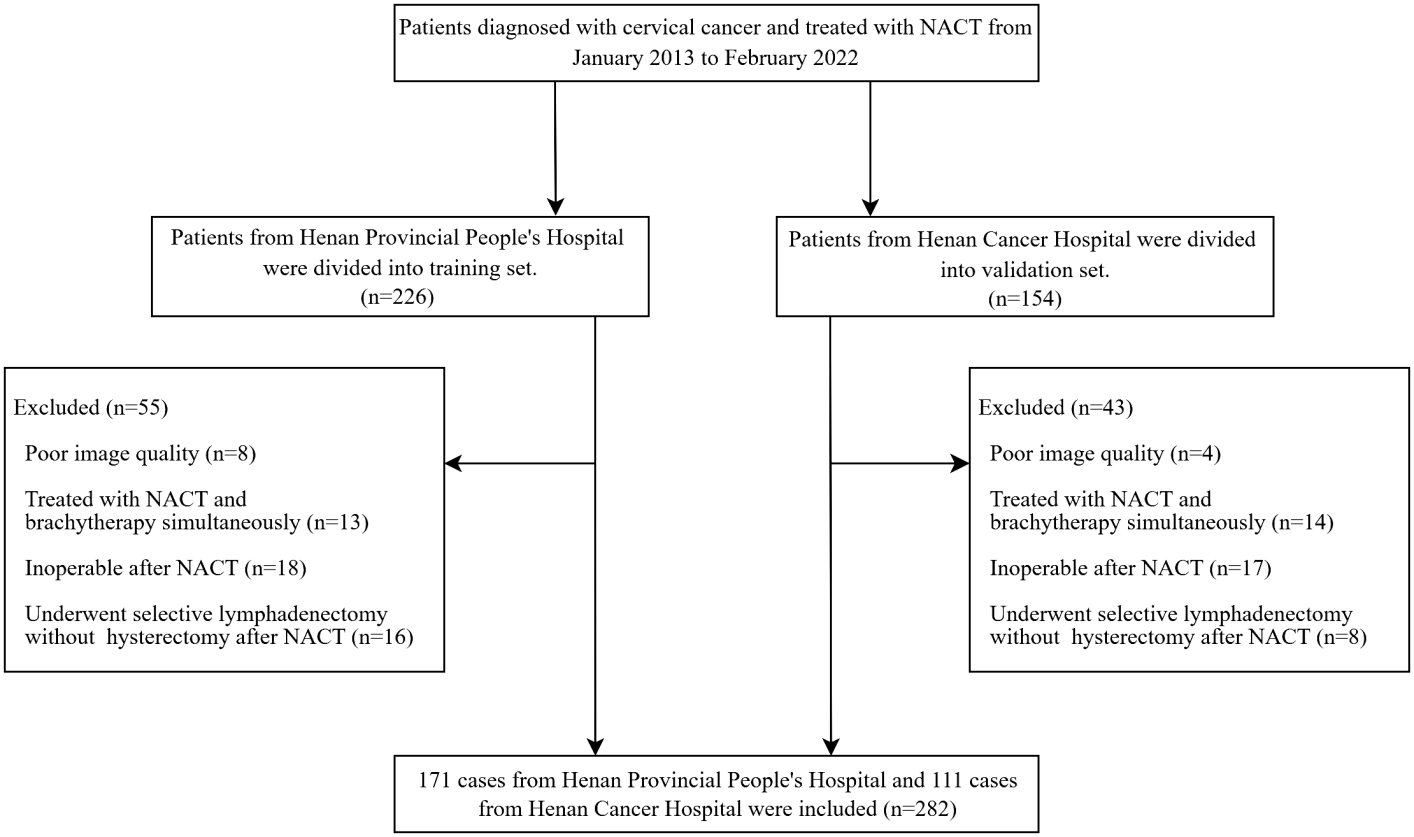
The patient recruitment flowchart.

Clinicopathological characteristics of patients, such as age, FIGO stage, NACT regimen, and tumor type were retrieved from their medical records. The LN status was used to adjust FIGO stage of the patients to conform with the 2018 staging system. Patients with LN (+) in MR images were classified as IIIC no matter what the original FIGO stage was (10). The LN was considered LN (+) if it met one of the following criteria: (a) a shortest diameter of more than 8mm; (b) ring enhancement showing necrosis inside the LN (23). The patient recruitment flowchart is shown in **Figure 1**.

### 2.2 Neoadjuvant chemotherapy protocol and follow-up

The NACT protocol included 1 to 3 cycles of platinum-based intravenous chemotherapy administered at three-week intervals. Gynecologists evaluated the feasibility of surgical resection and performed radical hysterectomy and lymphadenectomy three weeks after completion of NACT. If pelvic node disease was detected during intraoperative examination or if bulky aortic nodes were identified on preoperative imaging or during surgery, aortic lymphadenectomy was performed.

### 2.3 Lymph node involvement evaluation

All the resected surgical samples including the uterus and LNs were formalin fixed, paraffin embedded, and then histopathologically diagnosed by the pathologists of the two hospitals. LN status was extracted from the pathological reports.

### 2.4 MRI acquisition and image interpretation

Baseline MRI data (within 1 week before the NACT regimen started) were collected from two 3.0-T MR Platforms from Siemens Healthineers (MAGNETOM TrioTim 3.0 T, Skyra 3.0 T) and GE healthcare (discovery MR 750 3.0 T, signa HDxt 1.5 T). The MRI protocols of each hospital included sagittal T2-weighted imaging (Sag-T2WI) sequence, axial diffusion-weighted imaging (Ax-DWI) sequence, and the delayed phase of dynamic-contrasted-enhanced sagittal T1-weighted imaging (Sag-T1C) sequence. The comprehensive parameters of MR image acquisition of each hospital are detailed in **Supplementary Table S1**.

All images were analyzed independently by two radiologists, who were blinded to LNM results.

### 2.5 Tumor segmentation

The pretreatment MRI sequences were collated for tumor segmentation and feature generation. The regions of interest (ROI) were manually delineated on the cervical tumor slice by slice using ITK-SNAP (version 3.8.0 software: www.itksnap.org) containing the tumor mass on Sag-T2WI, Ax-DWI and Sag-T1C. To ensure accurate tumor boundaries, ROIs on all slices were carefully delineated manually by a radiologist with 13 years of experience in abdomen and pelvis imaging, who was blinded to the pathological findings.

Then, the ROI-based features of images from 30 randomly selected patients were re-extracted to test the features’ reproducibility for extraction in a blind fashion by the same radiologist. Only features with ICCs >0.75 were considered stable and selected for subsequent analysis.

### 2.6 Extraction and selection of radiomics features

The process of feature extraction and selection was carried out using the in-house software (PyRadiomics package version 2.3.0). First, all images were normalized using the max–abs scaling algorithm. Second, resampling and interpolation were also applied. The Sag-T1C images were reconstructed to 1mm×1mm×1mm voxel size, the Ax-DWI and Sag-T2WI images were reconstructed into 1mm×1mm×4mm image voxel sizes using a neural network-based B-spline surface construction approach to convert 2D ROI to 3D volume of interest (VOI). A total of 2264 features were extracted from each labeled ROI, including first order statistics features, shape-based (2D and 3D) features, gray level co-occurrence matrix (GLCM) features, gray level dependence matrix (GLDM) features, neighborhood gray tone difference matrix (NGTDM) features, gray level run-length matrix (GLRLM) features, and gray level size zone matrix (GLSZM) features.

After 3 months, 40 patients [30 LN (-) and 10 LN (**+**)] were randomly selected and resegmented by the same radiologist (QW) to assess intra-observer reliability. Only features with intra-observer ICCs > 0.75 were considered stable and selected for subsequent analysis.

The z-score standardization method was used for feature normalization. The K-Best method was first used to remove features with low correlation with classification labels in order to reduce computational complexity and prevent overfitting. And then LASSO was used to remove redundant features. Finally, the radiomics features significantly correlated with LNM of LACC were selected.

### 2.7 Model development and validation

The Sag-T2WI model, DWI model, and Sag-T1C model were constructed based on the selected radiomics features from Sag-T2WI, Ax-DWI, and Sag-T1C sequence, respectively. In order to build a more individualized predictive model, the correlation analysis was performed among all features of the three sequences, and the highly correlated features (r >0.75) were excluded, then the selected features were fused with clinical characteristics to construct a combined model. All of the models were developed using random forest classifier based on the training dataset, and the performance of which were evaluated and compared in the validation dataset.

**Figure 2:**
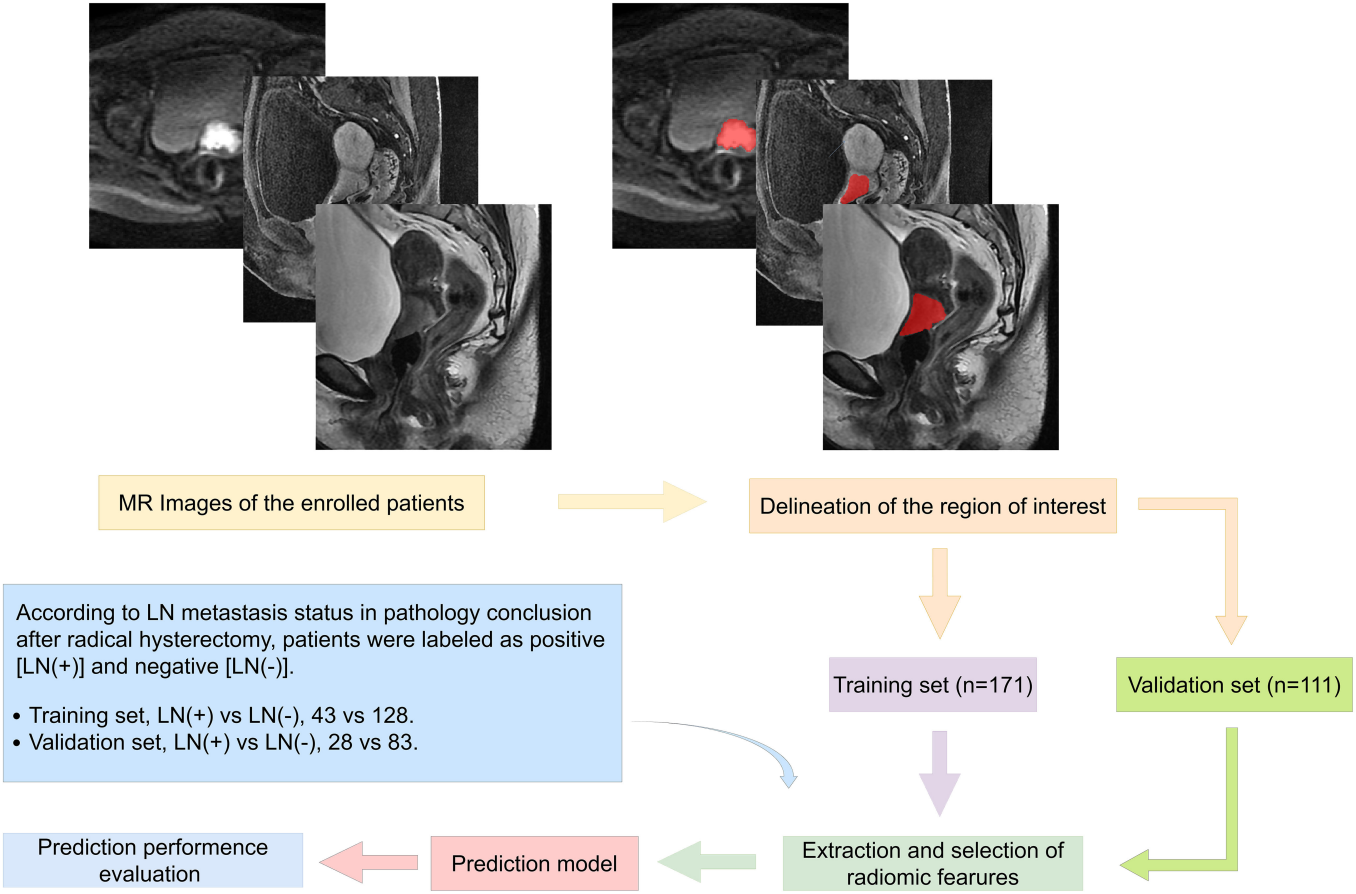
Study framework, including ROI segmentation and processing, radiomics feature extraction, feature selection, prediction signature establishment, and performance evaluation.

The development and validation of models were performed with InferScholar platform version 3.5 (InferVision, Beijing, China). The study framework is shown in **Figure 2**.

### 2.8 Statistics

Statistical analyses were performed using IBM SPSS software (version 28.0, Chicago, IL, USA). The differences between continuous clinical variables were evaluated by the Mann Whitney U test. The spearman correlation analysis was used to measure the correlation between arbitrary features. The visualization of the features used to build the combined model is illustrated using heatmap drawn by the R language with “ComplexHeatmap” package. The ROC and DCA were plotted using the R language with the “rms” package (version 4.2.1) and the “rmda” package (version 1.6), respectively. Delong’s test was used to compare the differences among AUCs of different models and different MRI instruments. The Hosmer-Lemeshow test was used to evaluate the consistency between the predicted and actual positive rate of LN in the validation set. A two-sided P <0.05 was considered statistically significant.

## 3 Results

### 3.1 Patient characteristics

The study included 282 patients diagnosed with LACC. The training set comprised 171 patients from HPPH, consisting of 43 LN (+) and 128 LN (-) cases. Simultaneously, the validation set involved 111 patients from HCH, with 28 LN (+) and 83 LN (-) cases. **Table 1** presents the clinical characteristics of the patients. Importantly, only the FIGO stage showed statistical significance between the LN (+) and LN (-) groups in both datasets (P < 0.001).

**Table 1:** Clinical characteristics.

Characteristics	Training set			Validation set		
	LN+ (n=43)	LN- (n=128)	P value	LN+ (n=28)	LN- (n=83)	P-value
Age, n (%)			0.018			0.975
≤50 years	25 (14.6%)	48 (28.1%)		7 (6.3%)	21 (18.9%)	
>50 years	18 (10.5%)	80 (46.8%)		21 (18.9%)	62 (55.9%)	
NACT regimen, n (%)			0.688			0.593
1 cycle	12 (7%)	31 (18.1%)		4 (3.6%)	10 (9%)	
2 cycles	27 (15.8%)	79 (46.2%)		22 (19.8%)	61 (55%)	
3 cycles	4 (2.3%)	18 (10.5%)		2 (1.8%)	12 (10.8%)	
Tumor type, n (%)			0.987			0.497
SCC	38 (22.2%)	113 (66.1%)		26 (23.4%)	71 (64%)	
Non-SCC	5 (2.9%)	15 (8.8%)		2 (1.8%)	12 (10.8%)	
FIGO stage			< 0.001			< 0.001
IB-IIB	19 (11.1%)	107 (62.6%)		2 (1.8%)	55 (49.5%)	
IIIA-IIIC	24 (14%)	21 (12.3%)		26 (23.4%)	28 (25.2%)	

LN+, positive lymph node metastasis; LN-, negative lymph node metastasis; NACT, neoadjuvant chemotherapy NACT; SCC, squamous cell carcinoma; Non-SCC, Non-squamous cell carcinoma; FIGO, International Federation of Gynecology and Obstetrics.

### 3.2 Selection of radiomics features and clinical factors

**Figure 3:**
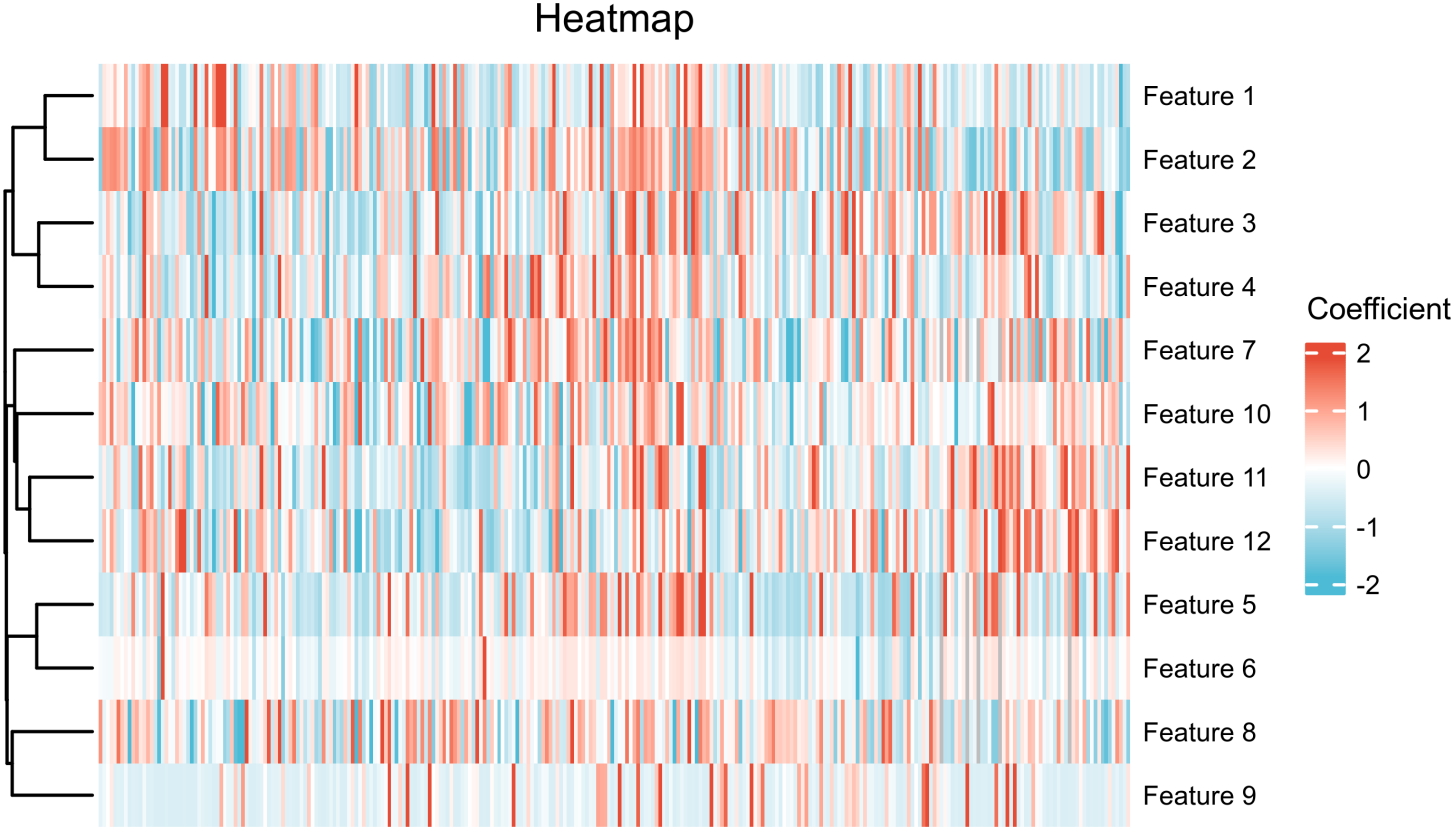
Heatmap of features included in combined model. The feature names represented by each serial number are annotated in **Table 2**.

A total of 5 Ax-DWI features (5 textural features), 4 Sag-T1C features (4 textural features) and 4 Sag-T2WI features (1 first order feature and 3 textural features) were selected (**Table 2**). And these features were utilized to construct three single-sequence models, respectively. Spearman correlation analysis was used to eliminate redundant features in the following way: when a pair of features showed a coefficient >0.75, the feature with the lower P value was remained. As a result, the Feature 13, wavelet_glcm_wavelet-LHL-Autocorrelation, was excluded. Subsequently, 4 Ax-DWI features, 4 Sag-T1C textural features, 1 Sag-T2WI first order feature, and 3 Sag-T2WI textural features were employed to construct the combined model (**Figure 3**). According to Mann Whitney U test, only FIGO stage (P <0.001) used for the construction of the combined model.

**Table 2:** Radiomics features for prediction models.

Model	Feature serial number	Feature name
Sag-T2WI model	Feature 1	additivegaussiannoise_firstorder_RootMeanSquared
	Feature 2	additivegaussiannoise_glszm_GrayLevelNonUniformityNormalized
	Feature 3	log_glrlm_log-sigma-4-0-mm-3D-LongRunEmphasis
	Feature 4	wavelet_glrlm_wavelet-LLH-RunVariance
Sag-T1C model	Feature 5	additivegaussiannoise_glrlm_LongRunLowGrayLevelEmphasis
	Feature 6	additivegaussiannoise_gldm_LargeDependenceLowGrayLevelEmphasis
	Feature 7	wavelet_glszm_wavelet-HLH-SmallAreaLowGrayLevelEmphasis
	Feature 8	normalize_glszm_SmallAreaEmphasis
Ax-DWI model	Feature 9	additivegaussiannoise_glszm_LargeAreaLowGrayLevelEmphasis
	Feature 10	wavelet_glcm_wavelet-LHL-ClusterTendency
	Feature 11	wavelet_glszm_wavelet-LHL-SmallAreaLowGrayLevelEmphasis
	Feature 12	specklenoise_glrlm_ShortRunLowGrayLevelEmphasis
	Feature 13	wavelet_glcm_wavelet-LHL-Autocorrelation
Combined model	Feature 1-12	–

glszm: Gray Level Size Zone Matrix; glrlm: Gray Level Run Length Matrix; gldm: Gray Level Dependence Matrix; glcm: Gray Level Co-occurrence Matrix; H: high-pass wavelet filter; L: low-pass wavelet filter.

### 3.3 Comparison of performance among models

**Figure 4:**
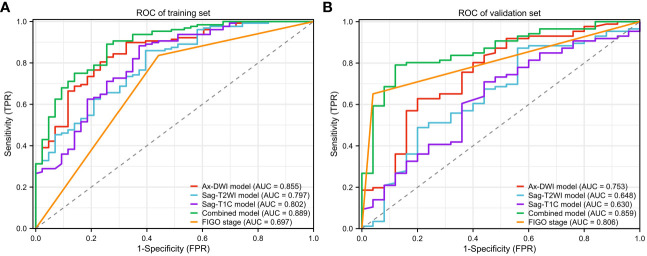
ROC of models (**(A)** ROC of training set. **(B)** ROC of validation set.).

Leveraging the remaining features, a combined model was developed, incorporating the FIGO stage. The ROC curves illustrating the performance are shown in **Figure 4**. The AUCs value, sensitivity, specificity, positive predictive value (PPV), and negative predictive value (NPV) of each model are summarized in **Table 3**.

**Table 3:** Diagnostic efficacy of single models and combined model.

Set	Model	AUC (95%CI)	Sensitivity	Specificity	PPV	NPV	Accuracy	Youden index
Training set	Sag-T1C model	0.802 (0.723-0.882)	0.882	0.628	0.876	0.643	0.819	0.573
	Sag-T2WI model	0.797 (0.722-0.872)	0.859	0.605	0.866	0.591	0.795	0.464
	Ax-DWI model	0.855 (0.791-0.919)	0.898	0.674	0.891	0.690	0.842	0.573
	FIGO stage	0.697 (0.615-0.779)	0.835	0.744	0.849	0.533	0.766	0.394
	Combined model	0.889 (0.833-0.945)	0.890	0.558	0.912	0.696	0.853	0.635
Validation set	Sag-T1C model	0.630 (0.505-0.756)	0.709	0.560	0.847	0.359	0.676	0.327
	Sag-T2WI model	0.648 (0.521-0.776)	0.872	0.440	0.843	0.500	0.775	0.312
	Ax-DWI model	0.753 (0.638-0.867)	0.627	0.800	0.915	0.384	0.667	0.427
	FIGO stage	0.806 (0.742-0.870)	0.651	0.880	0.982	0.444	0.811	0.611
	Combined model	0.859 (0.781-0.936)	0.791	0.960	0.958	0.550	0.721	0.671

Sag-T1C, sagittal T1-weighted contrast-enhanced imaging model; Ax-DWI, axial diffusion-weighted imaging model; Sag-T2WI, sagittal T1-weighted imaging model. PPV, positive predictive value; NPV, negative predictive value.

Notably, among the single-sequence models, the Ax-DWI model demonstrated the best predictive performance with AUCs of 0.855 (95%CI: 0.791-0.919) in the training set and 0.753 (95%CI: 0.638-0.867) in the validation set. There were no significant differences among AUCs of different single-sequence models in both sets (training set, Ax-DWI vs Sag-T2WI P =0.238, Ax-DWI vs Sag-T1C P=0.243, Sag-T2WI vs Sag-T1C P=0.921; validation set, Ax-DWI vs Sag-T2WI P =0.236, Ax-DWI vs Sag-T1C P=0.200, Sag-T2WI vs Sag-T1C P=0.838).

Furthermore, the diagnostic efficacy of the combined model, integrating radiomics features and the FIGO stage for LNM in LACC patients, surpassed that of among other models with AUCs of 0.889 (95%CI: 0.833-0.945) in the training set and 0.859 (95%CI: 0.781-0.936) in the validation set. However, according to the Delong test, only the difference between the combined model and the DWI model was not statistically significant in both the training (P =0.268) and validation sets (P =0.059). As shown in **Figure 4**, the model based on FIGO stage exhibited superior performance in the validation set with AUC of 0.806 (95%CI: 0.742-0.870) and displayed significant differences from the Sag-T2WI and Sag-T1C models (P <0.05).

Detailed comparisons between different models are presented in **Supplementary Table S2**. Additionally, no significant differences were observed among different MR image instruments (all P >0.05) (**Supplementary Table S3**).

### 3.4 Clinical utilities

**Figure 5:**
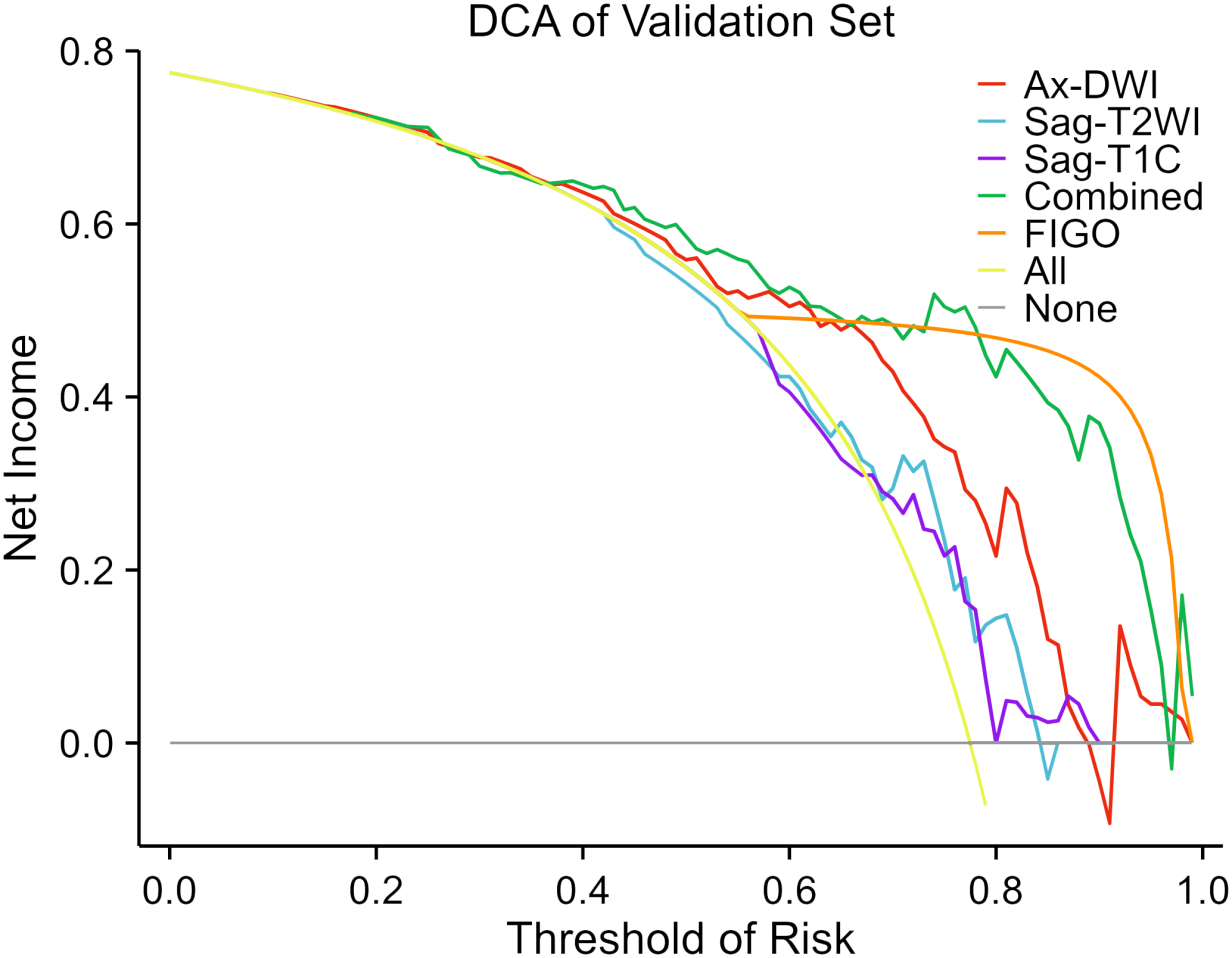
Decision curve analysis of the predictive models in the validation set. The decision curves reveal that if the threshold probability is over 40%, utilizing the combined model to predict LNM after NACT provides more benefit than the alternative strategies of treating all or none of the LACC patients.

The DCA of the prediction model in the validation set is depicted in **Figure 5**. If the threshold probability is below 80%, the combination model exhibited a higher net benefit compare to the FIGO stage and other single-sequence models in distinguishing LN (+) from LN (-).

### 3.5 Consistency test

The Hosmer-Lemeshow test indicated that in the validation set, all models showed good agreement between the predicted LNM probability and the observed incidence (Sag-T1C model, P =0.904; Ax-DWI model, P =0.078, Sag-T2WI model, P =0.127; Combined model, P =0.720).

## 4 Discussion

This study developed and validated a combined model based on three single-sequence MRI radiomic features in conjunction with the FIGO stage for pretreatment prediction of LNM in patients with LACC who underwent NACT. The combined model demonstrated superior performance compared to single-sequence-based radiomics with higher efficacy, which holds clinical significance for optimizing therapeutic strategies by the stratifying patients according to the benefit from NACT.

Radiomics features encompass first-order, shape-based, and textural features. Each feature dimension signifies distinct tumor information. The first order features describe the distribution of voxel intensities within the image region defined by the mask. The textural features show the gray value changes, which reflect the characteristics of intra-tumoral heterogeneity (25, 26). Several studies predicting LNM in malignant tumors, based on radiomics features, have screened out textural features (21, 22, 27, 28). A total of 12 textural features and 1 first-order feature were finally selected in our study. It’s noteworthy that in Hou L’s study on cervical cancer LNM using preoperative radiomics features, shape-based features were not screened out (21). Similarly, our study did not filter out shape-based features. This implies that the tumor shape characteristics of LACC may not exhibit a significant correlation with LNM.

In Hou L’s study on constructing cervical cancer LNM prediction models using preoperative radiomics features, within the single-sequence models, the DWI model demonstrated superior performance in predicting LNM in cervical cancer compared to the T2WI and enhanced T1WI models in the training set. However, this advantage was not as evident in the validation set (21). In contrast, our study revealed that the model based on Ax-DWI features exhibited superior diagnostic performance compared to Sag-T2WI and Sag-T1C models, achieving the highest AUCs in both sets. Although the differences between the Ax-DWI model and Sag-T2WI and Sag-T1C models were not statistically significant. The DWI sequence possess the capability to depict crucial information such as water fluidity, histiocytosis, and cell membrane integrity (29). The information can be quantified through radiomics features extracted from the DWI sequence (20), demonstrating a stronger correlation with LNM.

Similar to other studies predicting LNM of cervical cancer based on preoperative MRI radiomics features, the combined model in our study demonstrated significantly better performance for LNM in LACC patients compared to the Sag-T2WI and Sag-T1C models (21, 22). This may be related to the fact that the combined model covers more comprehensive lesion characteristics compared to single-sequence models. Furthermore, the DCA curve indicated that if the threshold probability is below 80%, the combined model exhibited the highest net benefit compare to the other models in distinguishing LN (+) from LN (-). This suggests that when the risk of LNM is low, radiomics can enhance the detection rate of positive LN, thereby improving the sensitivity of LN diagnosis and reducing the rate of missed diagnosis. Song J’s study also confirmed the role of radiomics in distinguishing positive or negative normal-sized LNs that are challenging to identify using conventional MRI in patients with cervical cancer (22). However, when the risk of LNM is high, multi-sequence MR can identify the internal structural changes of LNs, and radiomics may not further improve the accuracy of LN diagnosis anymore (30). Moreover, the model based on FIGO stage exhibited the highest net benefit when the threshold probability is over 80% in our study.

The study has several limitations. Firstly, a prospective study from more centers with considerably large cohorts is necessary to further confirm the performance of our combined model. Secondly, the absence of MR imaging after NACT prevents us from assessing changes in LNs between pre- and post- treatment. Thirdly, imaging data of LACC patients receiving chemoradiotherapy were not analyzed in this study, so the applicability of our model is limited. Finally, this retrospective study may be subject to inevitable selection bias. We aim to develop novel models utilizing LN radiomics features.

## 5 Conclusion

The present study applied radiomics features to predict LNM in patients with LACC. The experimental findings demonstrate that the model proposed in this study holds potential clinical value for enhancing the diagnostic efficiency of LNM in LACC patients, thereby impacting early non-invasive diagnosis and treatment planning for LNM in cervical cancer. It is anticipated to serve as an adjunctive tool for assessing LNM in LACC patients prior to NACT.

## Data availability statement

The raw data supporting the conclusions of this article will be made available by the authors, without undue reservation.

## Ethics statement

The studies involving human participants were reviewed and approved by the Institutional Review Board of Henan Province People’s Hospital and Henan Cancer Hospital. The studies were conducted in accordance with the local legislation and institutional requirements. Written informed consent from the patients/participants or patients/participants’ legal guardian/next of kin was not required to participate in this study in accordance with the national legislation and the institutional requirements.

## Author contributions

JL: Data curation, Formal analysis, Software, Visualization, Writing – original draft, Writing – review & editing. LD: Data curation, Formal analysis, Writing – review & editing. XZ: Data curation, Methodology, Conceptualization, Formal analysis, Project administration, Validation, Investigation, Visualization, Writing – original draft, Writing – review & editing. QW: Data curation, Methodology, Formal analysis, Software, Visualization, Writing – original draft, Writing – review & editing. ZY: Data curation, Formal analysis, Writing – review & editing. YZ: Data curation, Formal analysis, Writing – review & editing. CX: Data curation, Methodology, Formal analysis, Validation, Investigation, Resources, Writing – review & editing. QW: Conceptualization, Data curation, Funding acquisition, Methodology, Project administration, Resources, Supervision, Validation, Writing – review & editing. MW: Conceptualization, Funding acquisition, Resources, Writing – review & editing.
