# Supplementary material for: Radiomics analysis for prediction of lymph node metastasis after neoadjuvant chemotherapy based on pretreatment MRI in patients with locally advanced cervical cancer
Source: Front Oncol. 2024 May 8;14:1376640. doi: 10.3389/fonc.2024.1376640 (PMC11109452; doi:10.3389/fonc.2024.1376640)
Supplement: Supplementary file 1 [file Table_1.docx]

Supplementary Material

# Supplementary Figures and Tables

## Supplementary Tables

**Supplementary Table S1.** MRI protocols of the two centers.

| **Hospital** | **Scanner** | **Sequence** | **TR (ms)** | **TE (ms)** | **Slice Thickness (mm)** | **Slice Gap (mm)** | **Bandwidth** | **Slices** |
| --- | --- | --- | --- | --- | --- | --- | --- | --- |
| **#1** | GE 3.0T  (Discovery MR 750) | Sagittal T2 | 4000 | 87 | 5 | 1 | 163 | 20 |
|  |  | Coronal T2 | 3927 | 81 | 5 | 1 | 122 | 20 |
|  |  | Axial T2 | 4167 | 87 | 5 | 2 | 139 | 22 |
|  |  | Axial T1 | 446 | 7 | 5 | 2 | 325 | 22 |
|  |  | Axial DWI | 2600 | 74 | 5 | 2 | 1953 | 22 |
|  |  | Axial T1 C+ | 4 | 2 | 2 | 0 | 558 | 108 |
|  |  | Sagittal T1C+ | 4 | 2 | 1.5 | 0 | 558 | 96 |
|  |  | Coronal T1C+ | 4 | 2 | 2 | 0 | 651 | 64 |
|  |  |  |  |  |  |  |  |  |
|  | SIEMENS 3.0T  MAGNETOM (TrioTim) | Sagittal T2 | 3000 | 96 | 4 | 1.2 | 250 | 21 |
|  |  | Coronal T2 | 4000 | 104 | 5 | 1 | 203 | 20 |
|  |  | Axial T2 | 4000 | 87 | 5 | 1.5 | 250 | 21 |
|  |  | Axial T1 | 700 | 11 | 5 | 1.5 | 203 | 21 |
|  |  | Axial DWI | 4500 | 76 | 5 | 1.5 | 1736 | 21 |
|  |  | Axial T1 C+ | 3.3 | 1.1 | 1.5 | 0 | 501 | 96 |
|  |  | Sagittal T1C+ | 3.3 | 1.2 | 2 | 0 | 501 | 96 |
|  |  | Coronal T1C+ | 3.3 | 1.1 | 1.5 | 0 | 501 | 96 |
|  |  |  |  |  |  |  |  |  |
| **#2** | GE 1.5T  (Signa HDxt) | Sagittal T2 | 5780 | 133 | 4 | 0.4 | 162.77 | 24 |
|  |  | Axial T2 | 6020 | 128 | 5 | 0.5 | 162.77 | 24 |
|  |  | Axial T1 | 520 | 7 | 5 | 0.5 | 244.14 | 24 |
|  |  | Axial DWI | 2250 | 64 | 5 | 0.5 | 1953.12 | 24 |
|  |  | Axial T1 C+ | 3.7 | 1.5 | 4 | 2 | 488.28 | 68 |
|  |  | Sagittal T1C+ | 3.3 | 1.5 | 2.4 | 1.2 | 325.51 | 88 |
|  |  |  |  |  |  |  |  |  |
|  | SIEMENS  3.0T  (Skyra) | Sagittal T2 | 5000 | 91 | 4 | 0.4 | 200 | 24 |
|  |  | Axial T2 | 4130 | 87 | 5 | 0.5 | 200 | 24 |
|  |  | Axial T1 | 600 | 21 | 5 | 0.5 | 520 | 24 |
|  |  | Axial DWI | 5190 | 59 | 5 | 0.5 | 960 | 24 |
|  |  | Axial T1 C+ | 6.1 | 2.9 | 3 | 0 | 520 | 48 |
|  |  | SagittalT1C+ | 6.3 | 3.0 | 2.5 | 0 | 520 | 44 |

### Supplementary Table S2. Comparisons between different models. The *Delong test* showed that, in the comparison between the combined model and the single-sequence models, only the difference between the combined model and the DWI model was not statistically significant in both the training and validation sets. And no significant differences were observed among different single-sequence models.

| Models | P value | |  |
| --- | --- | --- | --- |
|  | Training set | Validation set |  |
| The Ax-DWI model vs the Sag-T1C model | 0.243 | 0.200 |  |
| The Ax-DWI model vs the Sag-T2WI model | 0.238 | 0.236 |  |
| The Ax-DWI model vs the combined model | 0.268 | 0.059 |  |
| The Sag-T1C model vs the Sag-T2WI model | 0.921 | 0.838 |  |
| The Sag-T1C model vs the combined model | **0.008** | **0.002** |  |
| The Sag-T2WI model vs the combined model | **0.033** | **0.004** |  |
| The FIGO stage model vs the Ax-DWI model | **0.001** | 0.421 |  |
| The FIGO stage model vs the Sag-T1C model | 0.057 | **0.002** |  |
| The FIGO stage model vs the Sag-T2WI model | 0.070 | **0.023** |  |
| The FIGO stage model vs the combined model | **<0.001** | 0.099 |  |

### Supplementary Table S3. Analysis of differences among different MRI instruments.

| Instruments |  | The Ax-DWI model | |  | The Sag-T1C model | |  | The Sag-T2WI model | |  | The combined model | |
| --- | --- | --- | --- | --- | --- | --- | --- | --- | --- | --- | --- | --- |
|  |  | Z value | P value |  | Z value | P value |  | Z value | P value |  | Z value | P value |
| Training set  GE 3.0T (Discovery MR 750) vs SIEMENS 3.0T MAGNETOM (TrioTim) |  | 1.382 | 0.167 |  | 0.287 | 0.774 |  | 0.443 | 0.658 |  | 0.157 | 0.875 |
| Validation set  GE 1.5T (Signa HDxt) vs SIEMENS 3.0T (Skyra) |  | 0.761 | 0.447 |  | 1.689 | 0.091 |  | 0.781 | 0.453 |  | 0.334 | 0.738 |
